# Ancient mitogenomics elucidates diversity of extinct West Indian tortoises

**DOI:** 10.1038/s41598-021-82299-w

**Published:** 2021-02-09

**Authors:** Christian Kehlmaier, Nancy A. Albury, David W. Steadman, Eva Graciá, Richard Franz, Uwe Fritz

**Affiliations:** 1 Museum of Zoology, Senckenberg Dresden, A. B. Meyer Building, 01109 Dresden, Germany; 2 National Museum of The Bahamas, Marsh Harbour, Abaco, Bahamas; 3 Florida Museum of Natural History, University of Florida, Gainesville, FL 32611 USA; 4 Ecology Area, Department of Applied Biology, Miguel Hernández University, Av. de la Universidad, Torreblanca, 03202 Elche, Spain

**Keywords:** Biodiversity, Evolution, Palaeontology, Phylogenetics, Taxonomy

## Abstract

We present 10 nearly complete mitochondrial genomes of the extinct tortoise *Chelonoidis alburyorum* from the Bahamas. While our samples represent morphologically distinct populations from six islands, their genetic divergences were shallow and resembled those among Galápagos tortoises. Our molecular clock estimates revealed that divergence among Bahamian tortoises began ~ 1.5 mya, whereas divergence among the Galápagos tortoises (*C. niger* complex) began ~ 2 mya. The inter-island divergences of tortoises from within the Bahamas and within the Galápagos Islands are much younger (0.09–0.59 mya, and 0.08–1.43 mya, respectively) than the genetic differentiation between any other congeneric pair of tortoise species. The shallow mitochondrial divergences of the two radiations on the Bahamas and the Galápagos Islands suggest that each archipelago sustained only one species of tortoise, and that the taxa currently regarded as distinct species in the Galápagos should be returned to subspecies status. The extinct tortoises from the Bahamas have two well-supported clades: the first includes one sample from Great Abaco and two from Crooked Island; the second clade includes tortoises from Great Abaco, Eleuthera, Crooked Island, Mayaguana, Middle Caicos, and Grand Turk. Tortoises belonging to both clades on Great Abaco and Crooked Island suggest late Holocene inter-island transport by prehistoric humans.

## Introduction

**Figure 1:**
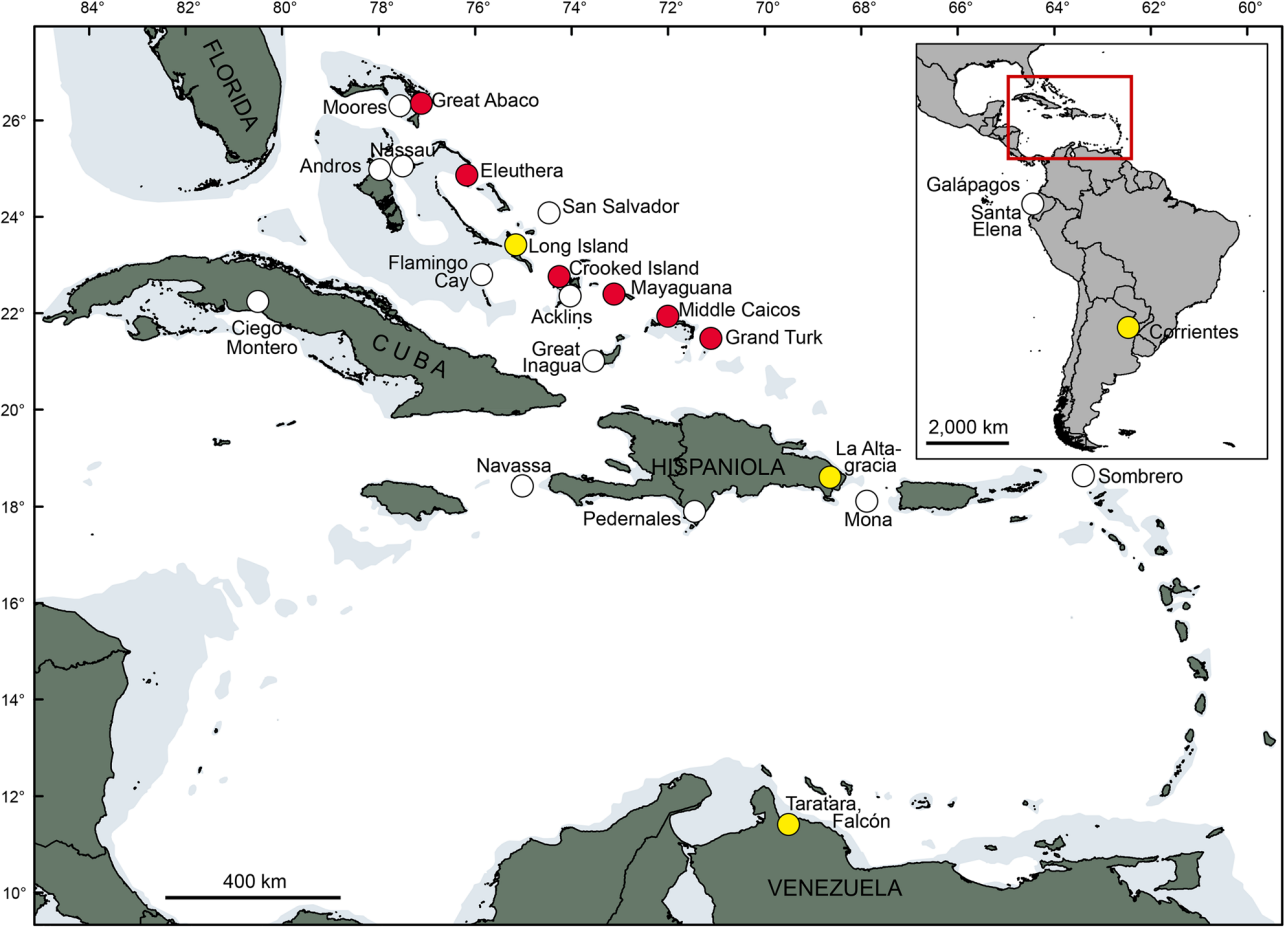
Late Quaternary and Holocene records of large-bodied or giant tortoises (*Chelonoidis* spp.) in South America and the Caribbean. Red rectangle in inset map corresponds to enlarged map sector. Open circles: unstudied material^5,6,9–11,21^, yellow circles: samples unsuccessfully processed for aDNA, red circles: samples successfully processed for aDNA. Grey areas show the shelf to a depth of 200 m. Map was created using ArcGIS 10.4 (https://www.esri.com/en-us/arcgis/about-arcgis/overview) and Adobe Illustrator CS6 (http://www.adobe.com/products/illustrator.html).

Giant tortoises (order Testudines, family Testudinidae) are charismatic reptiles, well known to naturalists, nature lovers, and the general public. Today, giant tortoises are restricted to the Galápagos Islands and the Aldabra Atoll. Little known is that giant tortoises were exterminated less than 200 years ago on the Mascarene Islands^1^. Unknown even to many zoologists is that large-bodied or giant tortoises roamed on many other oceanic islands during the Holocene and before^2^. Even in North and South America, giant tortoises occurred until the latest Pleistocene or early Holocene^3–10^. As a general rule, these large tortoise species disappeared after the arrival of humans, most likely as the result of unsustainable harvest^2^. One example is the tortoise radiation of the West Indies (Fig. 1).

No endemic tortoises survive today on these islands. What we know about the extinct tortoises is derived only from late Quaternary and Holocene fossils, which have been reported from the Bahamian Archipelago, Cuba, Hispaniola, Navassa, Sombrero, and Mona. These large tortoises fall into two major morphological groups, with the tortoises from the Bahamian islands (*Chelonoidis alburyorum*) distinguished from those on other West Indian islands, as well as the South American mainland, by lacking an epiplastral shelf^11^. Traditional morphological studies of West Indian tortoise fossils^12–15^ were greatly enhanced in 2004 by the discovery of superbly preserved tortoise fossils in Sawmill Sink, Great Abaco, Bahamas, which revealed morphological features impossible to discern in the fragmentary fossils previously available^16^. Furthermore, the Holocene fossils from Sawmill Sink retained enough collagen to allow radiocarbon dating and stable isotope analyses^17,18^. It followed, therefore, that the Sawmill Sink fossils should be assessed for ancient DNA (aDNA), which was successfully extracted and sequenced by Kehlmaier et al.^19^, allowing their firm phylogenetic placement in the same clade as the giant tortoises from Galápagos (*Chelonoidis niger* complex) and a small to medium-sized species from South America (*C. chilensis*).

Over the past decade, we have discovered tortoise fossils from a variety of late Quaternary localities (flooded sinkholes, dry sinkholes and caves, and archaeological sites) on 14 different islands in the Bahamian (Lucayan) Archipelago (Fig. 1). Many of these fossils are Holocene in age (< 10 ka) and retain enough collagen for radiocarbon dating and stable isotope analysis, just as had been done with the Sawmill Sink specimens^11,20^. In this paper, therefore, we build on the pioneering aDNA research of Kehlmaier et al.^19^ by expanding our coverage of tortoise aDNA across much of the island group. This information will allow us to evaluate the genetic relatedness of tortoises on different islands and different carbonate banks in the Bahamas. We also include late Quaternary material from sites in Argentina, Venezuela and the Dominican Republic (Hispaniola) with the aim to place the tortoises from the Bahamas in a comprehensive phylogenetic framework.

## Materials and methods

### Studied specimens

Seventeen subfossil bone samples of *Chelonoidis* were studied (Fig. 1), originating from the West Indies (*n* = 12) and mainland South America (*n* = 5), including material from the holotype of *C. dominicensis*^21^ from the Dominican Republic, a sample of the holotype of *C. lutzae*^7^ and three additional samples of *C.* cf. *lutzae* from Argentina, and one sample of an undescribed giant species of *Chelonoidis* from Venezuela^6^. The remaining specimens from the West Indies (*C. alburyorum*) originated on the Bahamian Islands (Great Abaco, Eleuthera, Long Island, Crooked Island, Mayaguana, Middle Caicos, and Grand Turk). Locality and collection details are provided in Table 1.

**Table 1:** The specimens of Bahamian tortoise (*Chelonoidis alburyorum*) with aDNA sequences reported in this paper.

Island, site	Type of site	Specimen	Laboratory number	Contig length [bp]	Average coverage	Accession number	^14^C Age (cal BP)
Crooked Island, 1702 Cave	DC, NC	NMB.CR026.3B	18985	15,349	110	LR968546	2740–2490
Great Abaco, Sawmill Sink	FS, NC	NMB.AB50.0008	–	15,328	–	LT599482*	970–920
Crooked Island, 1702 Cave	DC, NC	NMB.CR026.3A	18984	15,349	78	LR968545	2740–2490
Eleuthera, Kelly’s Blue Hole	FS, NC	NMB.EL180.27	18991	15,335	12	LR968551	–
Mayaguana, Abraham’s Bay Cave	DC, NC	NMB.MY014.3	18989	15,329	9	LR968549	–
Great Abaco, Lost Reel Cave	FS, NC	NMB.AB52.4	18994	15,346	11	LR968552	1230–1210, 1180–1060**
Grand Turk, Coralie	OS, C	GT-3, FS 82	18982	15,339	203	LR968543	903–846, 833–728**
Grand Turk, Coralie	OS, C	GT-3, FS 234	18987	15,349	482	LR968547	1179–1047, 1032–985**
Middle Caicos, Indian Cave	DC, NC	MC-37, Unit 9, surface	18983	15,350	276	LR968544	1060–930
Middle Caicos, Indian Cave	DC, NC	MC-37, Unit 4, II/7	18988	15,349	67	LR968548	1300–1240, 1200–1180**
Crooked Island, McKay’s Bluff Cave	DC, C/NC	NMB.CR05	18990	15,288	12	LR968550	925–785

The vertical sequence here matches the vertical sequence in the time tree. Type of site categories: *DC* dry cave, *FS* flooded sinkhole (blue hole), *OS* open site, *C* cultural, *NC* non-cultural. ^*14*^*C Age (cal BP)* radiocarbon age in calibrated years Before Present (2σ) from Steadman et al.^20^. Specimens are in the collection of the National Museum of The Bahamas (NMB), except for Grand Turk and Middle Caicos, which are in the Turks and Caicos National Museum and for which we provide field provenience. See Table S1 for further details.

*Sequenced in a previous study^19^.

**Two age ranges due to natural atmospheric variation through time in abundance of ^14^C because of sunspot activity; see Steadman et al.^20^.

### Processing of subfossil *Chelonoidis* samples

All samples were processed according to established guidelines^22^ in the aDNA facility of the Senckenberg Natural History Collections Dresden, which is physically separated from the main molecular genetic laboratory in another building. Negative controls (water blanks) were included during DNA extraction and library preparation and screened for evidence of contamination. Most bones were sampled using a Proxxon Micromot 50/E multitool equipped with 2–4 mm metal or stone drilling bits. Only the samples of *C. lutzae*/*C.* cf. *lutzae* were received as bone powder. Approximately 50 mg of bone powder of each sample was processed according to a protocol optimised for the recovery of short DNA fragments (Table S1)^23^. Then, up to 14 ng of DNA was converted into single-indexed, single-stranded Illumina sequencing libraries according to Gansauge and Meyer^24^ and Korlević et al.^25^, including the removal of uracil residues by uracil-DNA glycosylase (UDG) treatment. In order to increase the amount of endogenous mitochondrial DNA in the libraries, two-rounds of in-solution hybridization capture^26,27^ were performed in a dedicated capture-only workspace in the main laboratory using DNA baits generated from long-range PCR products of *C. carbonarius*, *C. chilensis*, *C. denticulatus*, and *C. vicina* at an equimolar rate. Details for long-range PCR, primer sequences, and PCR conditions are explained in the Supplementary Information. Sequencing was performed in-house on an Illumina MiSeq platform, generating 75 bp-long paired-end reads.

### Mitogenome sequence assembly and sequence annotation

Assembly of mitogenome sequences from the enriched libraries involved adapter trimming with Skewer 0.2.2^28^, read merging (minimum length 35 bp), quality filtering (minimum Q-score 20) and duplicate removal with BBmap-suite 37.24 (https://sourceforge.net/projects/bbmap/)^29^. The remaining reads were screened for contamination using FastQ Screen 0.11.4^30^ and a set of predefined mitochondrial genomes (Table S2). The identified non-target reads were excluded from the readpools, which are subsequently referred to as readpools 1. Then, all reads mapping to a reference genome of *Chelonoidis* were copied into a second readpool (readpools 2). Genome assembly was achieved with MITObim^31^, a two-step baiting and iterative mapping approach, with an allowed mismatch value of 2 and a starting seed according to Table S1. For each sample, readpool 2 was used for the initial building of a reference genome, whereas readpool 1 was used for the actual assembly. Resulting scaffolds were visualised and checked for assembly artefacts in Tablet^32^. Assembly artefacts were manually removed from the assembled contigs and all positions with a coverage below threefold masked as ambiguous (N) using the maskfasta subcommand of BEDTools 2.29.2^33^. Sequence length distribution of mapped reads was calculated with a customised awk command and Microsoft Excel.

### Alignment and phylogenetic, divergence time and biogeographic analyses

The newly generated mitogenomes of *Chelonoidis* were merged with our previously used annotated alignment^34^ for tortoises, with the exception that only one sequence per *Cylindraspis* species was included. This alignment also contained previously obtained data for a subfossil specimen of *Chelonoidis alburyorum* from Sawmill Sink, Great Abaco, Bahamas^19^. We also included 15 recently published sequences of *Chelonoidis* spp. from South America and Galápagos^35^ and removed a GenBank sequence for a Galápagos tortoise of unknown provenance (accession number JN999704) from the original alignment^34^.

To briefly summarise the approach, an automated preliminary alignment was generated using Clustal W 1.4^36^ and default parameters, as implemented in BioEdit 7.0.9.0^37^. This alignment was adjusted manually, and sequences were annotated using MITOS^38^ and several published tortoise mitogenomes (GenBank/ENA accession numbers: AF069423, DQ080042, DQ080048, FJ469674, KT613185, LT599485). Each coding region was screened for internal stop codons using MEGA X^39^. Finally, problematic sequence features (stop codons, gene overlap, frameshifts, spacer DNA) were removed (Supplementary Information).

Our final alignment of 15,516 sites comprised 66 sequences corresponding to all extant genera and species groups of tortoises (Testudinidae). It also included one representative each of the extinct Mascarene tortoise genus *Cylindraspis*^34^. The two outgroup taxa represented the successive sister taxa of Testudinidae, Geoemydidae (*Mauremys reevesii*) and Emydidae (*Chrysemys picta*).

Phylogenetic relationships of the mitogenomes were inferred with Maximum Likelihood (ML) and Bayesian Inference (BI) approaches using RAxML 8.0.0^40^ and MrBayes 3.2.6^41^. The best evolutionary models and partitioning schemes (Tables S3, S4) were determined with PartitionFinder2^42^ applying the greedy search scheme and the Bayesian Information Criterion. For ML, 20 independent searches were carried out using the GTR + G substitution model, different starting conditions, and the rapid bootstrap option. Subsequently, 1000 non-parametric thorough bootstrap replicates were calculated and the values plotted against the best tree. For BI, four parallel runs (each with eight chains) were performed with 2 million generations (burn-in 0.25; print frequency 1000; sample frequency 1000). Calculation parameters were analysed using Tracer 1.7.1^43^. In addition, uncorrected *p* distances were calculated in MEGA X^39^ using the pairwise deletion option. Divergence times were estimated using the uncorrelated lognormal relaxed clock models implemented in BEAST 1.84^44^ and constrained by four fossil calibration points following Kehlmaier et al.^34^ (Table 2). Further details for analyses are explained in the Supplementary Information.

**Table 2:** Calibration points (following Kehlmaier et al.^34^) used for the uncorrelated lognormal relaxed clock models implemented in BEAST.

Node	Mean	SD	Offset	2.5%-97.5% PDQs	Fossils
(A) Geoemydidae *–* Testudinidae	25.4	0.5	50.3	58.7–110	*Hadrianus majusculus*
(B) Testudinidae	16	0.5	33.9	39.2–71.5	*Cheirogaster maurini* and *Gigantochersina ammon*
(C) Testudininae	6	0.6	33.9	35.5–50.1	*Cheirogaster maurini*
(D) *Chelonoidis carbonarius – C. denticulatus*	10.75	0.5	11.8	15–36.8	*Chelonoidis hesternus*

Dates were set in million of years ago. For details, see Supplementary Information.

*PDQs* posterior distribution quantiles.

## Results

Ten of the 17 subfossil *Chelonoidis* samples produced high-quality data representing nearly the entire mitochondrial genome (15,288–15,350 bp length, coverage: ninefold to 482-fold; Table S1). This is an excellent yield for aDNA from tropical environments^19,45^, especially when it is considered that some specimens were from open unsheltered sites (Table 1).

For the mitogenomes, only the control region and part of adjacent DNA coding for tRNAs could not be reconstructed. All successfully assembled mitogenomes belonged to extinct tortoises from the Bahamas. The holotype of *Chelonoidis dominicensis* from the Dominican Republic, the material from Argentina (holotype of *C. lutzae*, three samples of *C.* cf. *lutzae*), the single species-undetermined *Chelonoidis* from Venezuela, and one *Chelonoidis* sample from Long Island, Bahamas, did not yield sufficient endogenous DNA. Assembly details of individual samples and blanks as well as genetic diversity indices and substitution rates for selected clades are provided in the Supplementary Information.

**Figure 2:**
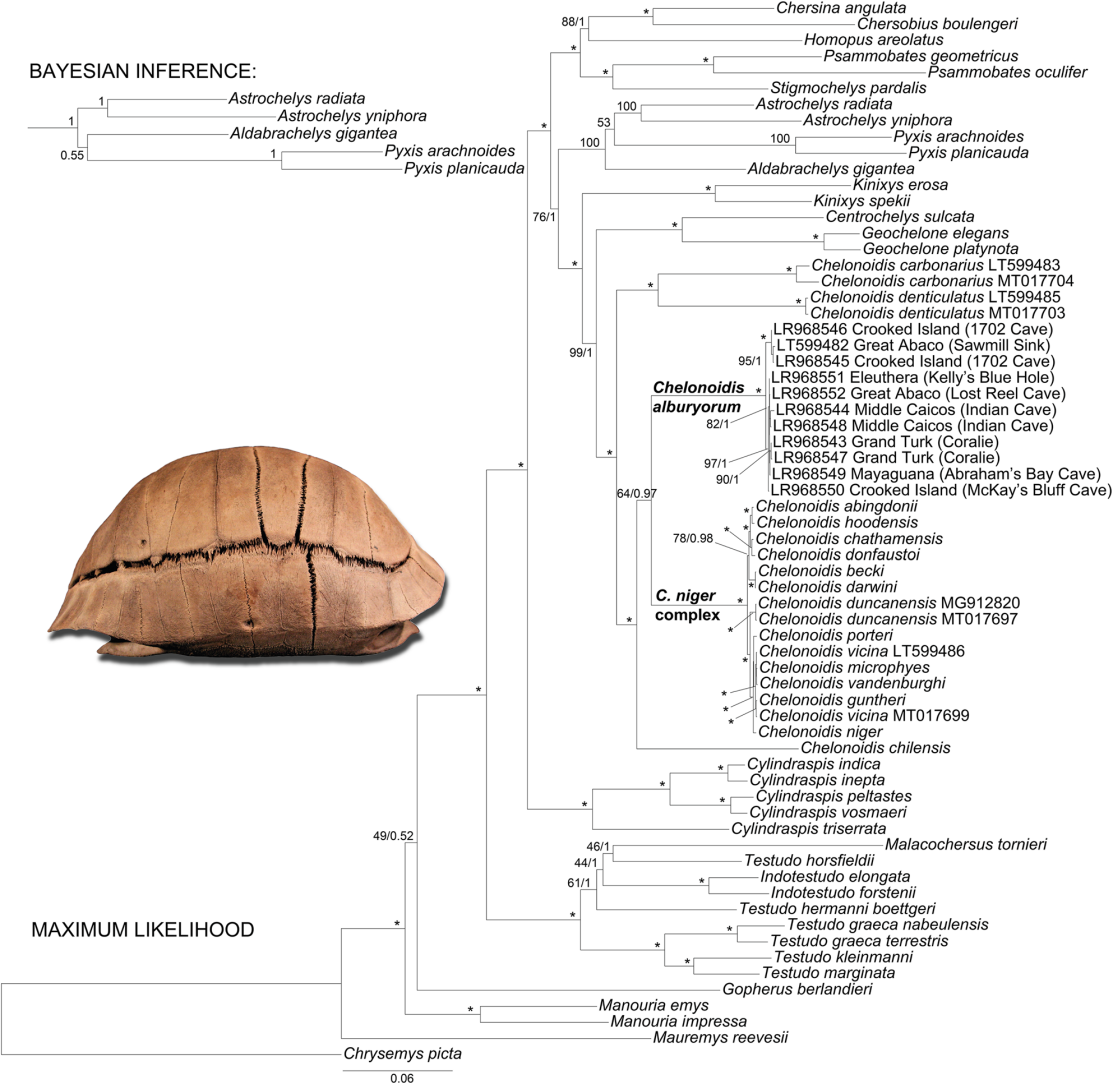
Maximum Likelihood tree for near-complete mitochondrial genomes of tortoises (15,516 bp). The tree includes living and extinct *Chelonoidis* species and representatives of all other extant genera and of the five recently extinct *Cylindraspis* species from the Mascarene Islands^34^. Ten of the 11 mitochondrial genomes of the Bahamian *Chelonoidis* specimens were produced in this study. Numbers at nodes are thorough bootstrap values and posterior probabilities from a Bayesian Inference tree; top left shows alternative Bayesian topology for the respective branches. Asterisks indicate maximum support under both tree-building approaches. Codes following species names or preceding localities are GenBank/ENA accession numbers (for remaining accession numbers, see Supplementary Information). Inset: *Chelonoidis alburyorum* (National Museum of The Bahamas, NMB.AB50.0008, Sawmill Sink, Great Abaco, LT599482^19^; photo: N. A. Albury).

**Figure 3:**
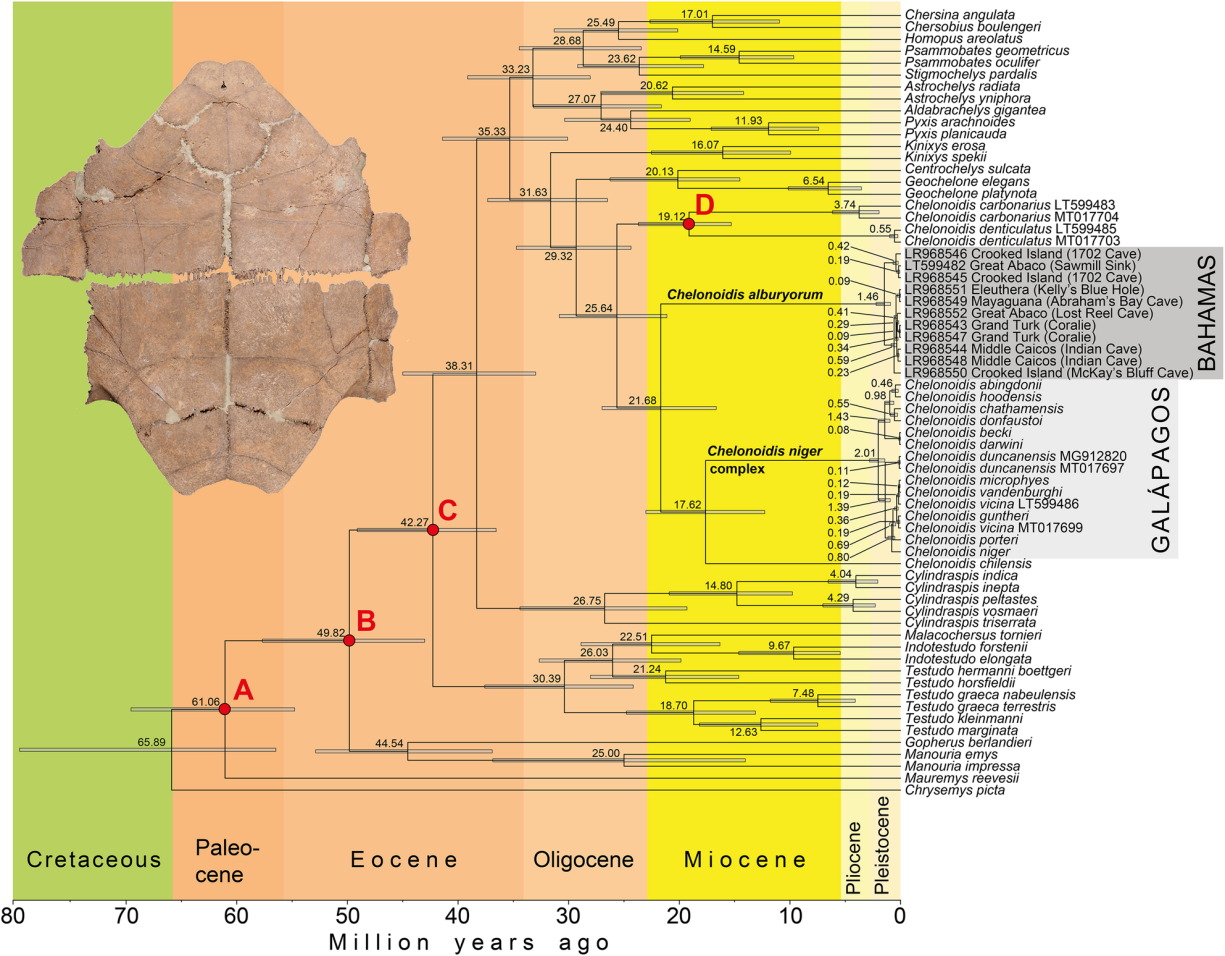
Fossil-calibrated time tree for near-complete mitochondrial genomes (15,516 bp) of all extant and some extinct tortoise genera. Codes following species names or preceding localities are GenBank/ENA accession numbers (for remaining accession numbers, see Supplementary Information). Inferred mean ages and 95% Highest Posterior Density intervals are shown for each node. The red circles indicate fossil-based constraints following Kehlmaier et al.^34^: (**A**) *Hadrianus majusculus*, 50.3–100.5 Ma; (**B**) *Cheirogaster maurini* and *Gigantochersina ammon*, 33.9–66.0 Ma; (**C**) *Cheirogaster maurini*, 33.9–47.8 Ma; (**D**) *Chelonoidis hesternus*, 11.8–33.9 Ma. For details of calibration, see Supplementary Information. Inset: Holotype of *Chelonoidis alburyorum keegani* (Florida Museum of Natural History, UF 453000, plastron, Coralie, Grand Turk, same site as sequences LR968543 and LR968547; photo: N. A. Albury).

Our phylogenetic analyses and the molecular clock calculation, including the 10 new and one previously published mitogenomes of West Indian *Chelonoidis* and additional data for *Chelonoidis* species from Galápagos and South America, produced general tree topologies consistent with our previous studies^19,34^. Of particular interest are the relationships of the crown clade containing *C. chilensis* and the *Chelonoidis* species from the Bahamas and Galápagos. These taxa were placed in a maximally supported clade, although the branching pattern within that clade was weakly resolved. Accordingly, the ML and BI trees suggested that *C. chilensis* is sister to a weakly supported clade consisting of the two island clades (Fig. 2), whereas our time tree reflected the weakly supported topology of our previous studies, with *C. chilensis* as sister taxon of the Galápagos tortoises (Fig. 3). Thus, the divergence time inferred for this node should be taken with caution. In contrast, the two clades containing giant tortoises from the Bahamas and Galápagos were both maximally supported (Fig. 2).

Our 10 new samples from the Bahamas, and the previously sequenced specimen^19^, represented morphologically highly divergent populations from six islands^11,16^. Nevertheless, the genetic divergences were shallow and resembled those among Galápagos tortoises (Fig. 2). Our molecular clock estimated a mean of 2.34 × 10^–3^ substitutions per site per million years (95% HPD: 2.11 × 10^–3^–2.78 × 10^–3^). Divergence among tortoises from the Bahamas commenced approximately 1.5 million years ago (mya), while divergence among tortoises from the Galápagos Islands began approximately 2 mya (Fig. 3). Two samples of the congeneric species *Chelonoidis carbonarius* from South America were more different genetically (with a divergence time of ~ 3.7 mya), as were two other pairs of congeneric tortoises (the extinct *Cylindraspis indica* from Réunion and its sister species *C. inepta* from Mauritius, that diverged ~ 4 mya, and the sister species *C. peltastes* and *C. vosmaeri* from Rodrigues, which diverged ~ 4.3 mya). In contrast, the divergence between two samples of *Chelonoidis denticulatus* was shallow and estimated to be ~ 0.6 million years old.

The inter-island divergences of tortoises from within the Bahamas and within the Galápagos Islands are much younger (0.09–0.59 mya, and 0.08–1.43 mya, respectively) than the genetic differentiation between any other congeneric pair of tortoise species (in Figs. 2 and 3 from top to bottom: *Psammobates* (14.6 mya), *Astrochelys* (20.6 mya), *Pyxis* (11.9 mya), *Kinixys* (16.1 mya), *Geochelone* (6.5 mya), *Chelonoidis carbonarius* + *C. denticulatus* (19.1 mya), *Cylindraspis* (26.8–4.0 mya), *Indotestudo* (9.7 mya,) *Testudo* (21.2–7.5 mya), and *Manouria* (25.0 mya). The mitochondrial divergence of subspecies of *Testudo graeca* (7.5 mya) also resembles or exceeds that between many sister species.

Within the extinct tortoises from the Bahamas, there are two well-supported clades (Figs. 2, 3), each of which died out from ~ 900 to 700 years ago (Table 1). One clade includes sequences from one sample from Great Abaco and two tortoises from Crooked Island; the other clade contains sequences corresponding to other tortoise specimens from Great Abaco and Crooked Island, as well as ones from Eleuthera, Mayaguana, Middle Caicos, and Grand Turk. The Galápagos Islands have six or seven clades of tortoises with genetic divergences similar to those of the two Bahamian clades.

## Discussion

Our study provides evidence that the extinct tortoises from the Bahamas had diverged very little genetically, despite pronounced morphological differences^11,16^. For example, while all of the Bahamian tortoises were large, the one from Lost Reel Cave on Great Abaco was by far the largest, approaching if not matching in size the largest extant tortoises from the Galápagos and Aldabra. The various Bahamian forms also displayed major differences (not age-related) in the shape, rugosity, and relative size of the entoplastron, epiplastron, hypoplastron, and xiphiplastron. It was upon these differences that Steadman et al.^20^ tentatively recognized that as many as seven distinct species of Bahamian tortoises existed. Subsequently, two of these forms were described as subspecies of *Chelonoidis alburyorum*, namely *C. a. sementis* from Middle Caicos and *C. a. keegani* from Grand Turk^11^ (Table S5).

According to our molecular clock, the Bahamian radiation commenced approximately 1.5 mya, i.e., about 500,000 years after the onset of the radiation of the giant tortoises on the Galápagos Islands. Another recently published estimate for the age of the Galápagos tortoise radiation is slightly younger than ours (1.5 mya instead of 2.0 mya)^35^, resembling our molecular clock results for the Bahamas.

The Galápagos Islands formed approximately 4 mya^46^, whereas the age of the Bahamas, from the standpoint of being able to support terrestrial vertebrates, is estimated not to exceed 400,000 years because of interglacial flooding during marine isotope stage 11^47–49^. This situation implies for the Galápagos Islands that the current diversity of giant tortoises resulted from a single colonization event and a local radiation on the archipelago^35^. In contrast, the two tortoise clades from the Bahamas seem to be too old for having diverged on the islands. This suggests that the Bahamas may have been colonized twice from other landmasses. If that was the case, then the two colonizers must have been very similar genetically, given the low amount of genetic divergence of tortoises across the island group. As far as known, the extinct giant tortoises from the Greater Antilles, which would seem to be the likely source region of the Bahamian tortoises, are morphologically clearly distinct^11,21^. We cannot exclude, however, that these differences reflect morphological plasticity, a phenomenon well known from many other tortoise taxa^50–53^. Alternatively, the ancestral taxa on the Greater Antilles may still be undiscovered or known only by material too fragmentary to discern the crucial morphological characters. (The majority of tortoise fossils from the Greater Antilles are represented only by very incomplete material^21^). Unfortunately, the only sample from the Greater Antilles that we studied (ulna from the holotype of *C. dominicensis*, radiocarbon-dated to the early Holocene) did not yield aDNA sequences, so that genetic evidence must await further investigation. Nevertheless, *C. dominicensis* remains valuable for morphological studies, such as its possession of an epiplastral shelf, which is characteristic of the Galápagos and South American clades of *Chelonoidis* but not the Bahamian clade^21^.

An unexpected result of our study was that we found tortoises belonging to the two Bahamian clades on the same islands. One of these clades was represented only by two tortoises from Crooked Island (1702 Cave) and the previously sequenced specimen from Great Abaco (Sawmill Sink)^19^. The two specimens from Crooked Island are ~ 2600 years old, and therefore pre-cultural, whereas that from Abaco is ~ 950 years old (Table 1). Human arrival in the Bahamas took place ~ 1200 to 1000 years ago^17,20,54,55^. This situation suggests prehistoric human transport of tortoises from Crooked Island to Great Abaco, which lies on a different (and distant) bank.

The second Bahamian clade contained one specimen each from these same two islands, namely from McKay’s Bluff Cave on Crooked Island, and from Lost Reel Cave on Great Abaco, as well as specimens from Eleuthera, Mayaguana, Middle Caicos, and Grand Turk. This yields a total of six islands on six different banks (Fig. 1). Because the late Holocene specimens post-date the arrival of humans (Lucayans) in the Bahamas, the inter-island mixing of clades is strong evidence that early people were moving tortoises among islands, which could have also contributed to hybridization and an increase in morphological variation.

A similar situation was discovered recently with aDNA of the Bahamian hutia (*Geocapromys ingrahami*), again involving prehistoric human transport between Great Abaco and Crooked Island^56^. Whether tortoises or hutias, their inter-island transport by people did not prevent their eventual extinction on both Great Abaco and Crooked Island. We have no evidence that indigenous tortoises survived in the Bahamas beyond 800–700 cal BP^20^, which is several centuries before European contact.

In any case, compared to other tortoises (Figs. 2, 3; Table S6), the shallow mitochondrial divergences of the tortoise radiations on the Bahamas and the Galápagos Islands suggest that each archipelago harboured only one species and that the many taxa currently regarded as distinct species^1,35^ should be returned to subspecies status. This also is in agreement with the weak nuclear genomic divergence of Galápagos tortoises^57,58^.

Conspecificity is further supported when mitogenomic divergences within other *Chelonoidis* species are compared to those of the two island radiations. Two samples of the widely distributed South American species *C. carbonarius* were estimated in our molecular clock calculation to have diverged ~ 3.7 mya, and these samples differed by an uncorrected *p* distance of 1.7% compared to maximum values of 0.7% within the Bahamian radiation and 0.9% within the Galápagos radiation. The shallower divergence between two samples of another widely distributed South American species, *C. denticulatus* (~ 0.6 mya; 0.2% uncorrected *p* distance), is in line with a previous study^59^ that found the savannah species *C. carbonarius* more differentiated than its forest-dwelling sister species *C. denticulatus*.

Even though the genetic divergences among the tortoises of the Bahamas (and, for that matter, the Galápagos Islands) are small, each of the populations had a distinctive morphology with which it interacted in its environment. Both archipelagos have gradients of temperature and precipitation, yielding distinctive environments and vegetation types on different islands. Giant tortoises are known to play important roles for the vegetation structure and composition on other islands^60,61^, and undoubtedly once did the same on the Bahamas.

## Supplementary Information

Supplementary Information
