# Supplementary material for: Ancient mitogenomics elucidates diversity of extinct West Indian tortoises
Source: Sci Rep. 2021 Feb 9;11:3224. doi: 10.1038/s41598-021-82299-w (PMC7873039; doi:10.1038/s41598-021-82299-w)
Supplement: Supplementary file 1 — Supplementary Information [file 41598_2021_82299_MOESM1_ESM.pdf]

Ancient mitogenomics elucidates diversity of extinct West Indian tortoises

Christian Kehlmaier, Nancy A. Albury, David W. Steadman, Eva Graciá,
Richard Franz, Uwe Fritz

Scientific Reports
https://doi.org/10.1038/s41598-021-82299-w

Supplementary Information

Long-range PCRs for bait library preparation of the in-solution hybridisation capture approach

Long-range PCR reactions were performed in 50 µl volumes, containing 1–6 µl of high-molecular DNA extract and 1 unit of TaKaRa LA *Taq* DNA Polymerase, Hot-Start Version (Clontech Laboratories Inc., Mountain View, CA, USA), following the reaction mixture recommended by the manufacturer. Two pairs of primers, originally developed by Kehlmaier *et al*. (2017), were used: (i) Chelo_12S-ATP6_For (5'-GCRTACCAGTGAAAAYACCC-3'), Chelo_12S-ATP6_Rev (5'-TTGGGATRAGTGGGGTTGGG-3') and (ii) Chelo_ATP6-cytb_For (5'-AAGCCCACAAAYCCTWGGAA-3'), Chelo_ATP6-cytb_Rev (5'-GGATTAGGAGRATTGTGAAGT-3'). The combined long-range PCR products covered most of the mitochondrial genome from almost the beginning of 12S to almost the end of cyt *b*. PCR conditions comprised an initial denaturation at 93°C for 3 min, followed by 35 cycles of 93°C for 15 sec, 60°C for 30 sec, 68°C for 9 min, followed by a final elongation step at 68°C for 10 min.

Alignment details

After integrating the new mitochondrial sequences into a previously compiled alignment (Kehlmaier *et al*. 2019), totalling to 15,922 bp, certain positions were deleted prior to phylogenetic analyses: (1) stop codons of coding genes, as these do not code for any amino acid; (2) overlap between DNA coding for proteins or tRNAs, as these short regions cannot be attributed to a single gene and underlie a distinct evolutionary model; (3) alignment positions that cause frame shifts in coding regions; (4) non-coding spacer DNA, including an unalignable 141-bp-long non-coding stretch between ND5 and ND6 in *Chrysemys picta*. In total, 406 positions were deleted, resulting in a final alignment length of 15,516 bp.

Individual substitution rates and genetic diversity

Substitution rates (μ, substitutions per site per million years) were calculated manually for *Chelonoidis alburyorum* and two other lineages of giant tortoises, the closely related Galápagos tortoises (*C. niger* complex) and the distantly related extinct giant tortoises from the Mascarenes (*Cylindraspis* spp.). For doing so, the total number of available sites (i.e., without missing data/gaps; n_{sites}), the number of substitutions (S), and the diversification times inferred from the molecular clock (T) were used ($\mu = S/n_{\text{sites}}/T$). S and other general diversity indices were computed using the software DnaSP 6 (Rozas *et al*. 2017). For comparative purposes, these values were also obtained for *Chelonoidis carbonarius* and *C. denticulatus* and the two subspecies of *Testudo graeca* (Table S7).

Across all 11 mitogenomes of Bahamian *Chelonoidis* 9,841 sites in the alignment were without missing data/gaps. For these 9,841 sites, 63 substitutions occurred and no insertions or deletions (indels). The nucleotide diversity (π) was 0.002. These values do not compare completely to those of the other taxa, as there were more sites without missing data/gaps available. However, it may be parenthetically noted that the nucleotide diversities across the two *C. denticulatus* sequences (0.002) equalled those of the 11 *C. alburyorum*. The nucleotide diversities across the 15 Galápagos mitogenomes (0.006) were three times higher, and those of the two *C. carbonarius* (0.017), the 5 *Cylindraspis* spp. (0.06) and the two *T. graeca* subspecies (0.035) were higher by one order of magnitude. The mean substitution rates (μ) for all taxa except for the Galápagos tortoises ranged between 4.34 x 10^{-3} and 4.39 x 10^{-3}. The rate of the Galápagos tortoises was one order of magnitude higher (1.10 x 10^{-2}), which could be related to a rapid radiation.

Divergence time analyses

Molecular dating relied on the uncorrelated lognormal relaxed clock models implemented in BEAST 1.8.4 (Drummond *et al*. 2012). A Yule tree with the HKY substitution model and four rate categories was used. MCMC chains ran for 10^9 generations, with parameters and trees sampled every 10^4 generations. Tracer 1.7.1 (Rambaut *et al*. 2018) served to check for convergence of the runs using Effective Sample Sizes (ESS) of parameters after discarding 10% of the initial trees as burn-in (all statistics resulted in ESS > 200). Trees were summarized using TreeAnnotator 1.8.4 (Drummond *et al*. 2012) and the maximum clade credibility tree and mean node height options. Based on Kehlmaier *et al*. (2019), four nodes were calibrated using the following priors under lognormal distributions (letters refer to Fig. 3):

(A) The total clade of Testudinidae was constrained with a minimum at 50.3 Ma (i.e., the top of the Wasatchian North American Land Mammal Age) and a maximum at 100.5 Ma (i.e., the base of the Late Cretaceous) based on the unambiguous pantestudinid *Hadrianus majusculus* from sediments of the Wasatchian North American Land Mammal Age.

(B) The minimum age of Testudinidae was constrained at the top of the Eocene (33.9 Ma) using the late Eocene (Priabonian) *Cheirogaster maurini* and *Gigantochersina ammon*.

According to Vlachos & Rabi (2018), these taxa are deeply nested within Testudinidae. As maximum age of this clade, the base of the Tertiary (66.0 Ma) was used because no "tortoise," stem or crown, is known from the Mesozoic.

(C) The minimum age of Testudininae (i.e., all extant tortoises except *Manouria* and *Gopherus*) was constrained using the late Eocene (Priabonian) *C. maurini*. According to Vlachos & Rabi (2018) *C. maurini* is nested within the testudinine clade Geochelona. In contrast to other, potential, geochelonans from the late Eocene, *C. maurini* lacks a cervical scute, a character uniquely found in geochelonans among cryptodires. As maximum age 47.8 Ma was used because no tortoises with derived characters have been reported before the late Eocene.

(D) The divergence of the extant *Chelonoidis carbonarius* and *C. denticulatus* was calibrated using the fossil *C. hesternus* of Laventan South American Land Mammal Age. Our minimum of 11.8 Ma is based on the published minimum age for the Laventan, and our maximum of 33.9 Ma corresponds to the onset of the Oligocene, acknowledging that no tortoises have been reported from South America before the Oligocene (de la Fuente *et al*. 2018).

References

de la Fuente, M. S., Zacarías, G. G. & Vlachos, E. A review of the fossil record of South American turtles of the clade Testudinoidea. *Bull. Peabody Mus. Nat. Hist.* **59**, 269–286 (2018).

Franz, R., Albury, N. A. & Steadman, D. W. Extinct tortoises from the Turks and Caicos Islands. *Florida Mus. Nat. Hist. Bull*. **58**, 1–38 (2020).

Franz, R. & Franz, S. A new fossil land tortoise in the genus *Chelonoidis* (Testudines: Testudinidae) from the northern Bahamas, with an osteological assessment of other Neotropical tortoises. *Florida Mus. Nat. Hist. Bull.* **49**, 1–44 (2009).

Drummond, A. J., Suchard, M. A., Xie, D. & Rambaut, A. Bayesian phylogenetics with BEAUti and the BEAST 1.7. *Mol. Biol. Evol*. **29**, 1969–1973 (2012).

Kehlmaier, C. *et al*. Tropical ancient DNA reveals relationships of the extinct Bahamian giant tortoise *Chelonoidis alburyorum*. *Proc. R. Soc. B* **284**, 20162235 (2017).

Kehlmaier, C. *et al*. Ancient mitogenomics clarifies radiation of extinct Mascarene giant tortoises. *Sci. Rep*. **9**, 17487 (2019).

Rambaut, A., Drummond, A. J., Xie, D., Baele, G. & Suchard, M. A. Posterior summarization in Bayesian phylogenetics using Tracer 1.7. *Syst. Biol.* **5**, 901–904 (2018).

Rozas, J. *et al*. DnaSP 6: DNA sequence polymorphism analysis of large datasets. *Mol. Biol. Evol.* **34**, 3299–3302 (2017).

Steadman, D. W. *et al*. The paleoecology and extinction of endemic tortoises in the Bahamian Archipelago. *The Holocene* **30**, 420-427 (2020).

Vlachos, E. & Rabi, M. Total evidence analysis and body size evolution of extant and extinct tortoises (Testudines: Cryptodira: Pan-Testudinidae). *Cladistics* **34**, 652–683 (2018).

Table S1. Assembly details of samples and blanks. Vouchers are in the collection of Paleozoología Corrientes, Facultad de Ciencias Exactas, Naturales y Agrimensura, Universidad Nacional del Nordeste, Corrientes, Argentina (CTES-PZ), the National Museum of The Bahamas, Abaco, The Bahamas (NMB), and the Museo del Hombre Dominicano, Santo Domingo, Dominican Republic (MHD). Specimens from Grand Turk and Middle Caicos, for which we provide field provenience, are in the Turks and Caicos National Museum.

MTD lab number	Voucher	Taxon	Provenance	Age**	Dry bone powder into lysis	DNA conc. Qubit HS	DNA into ssLib prep	Raw reads	Quality filtered reads	MITObim assembled reads	MITObim seed	Average read length	Average coverage	Length of final contig submitted to ENA	Ambiguous sites in final contig
	**Samples that produced good sequence data**														
18982	GT-3, FS 82	*Chelonoidis alburyorum*	Grand Turk (Coralie)	903-846, 833-728	49 mg	1.78 ng/µl	13.0 ng	8,222,710	1,556,431	41,670	LT599482	74 bp	203	15,339 bp	160 bp
18983	MC-37, Unit 9, surface	*Chelonoidis alburyorum*	Middle Caicos (Indian Cave)	1060-930	50 mg	2.26 ng/µl	13.1 ng	3,354,289	1,422,600	55,214	LT599482	77 bp	276	15,350 bp	0 bp
18984	NMB.CR026.3A	*Chelonoidis alburyorum*	Crooked Island (1702 Cave)	2740-2490	47 mg	6.10 ng/µl	13.4 ng	5,988,849	2,304,598	14,537	LT599482	80 bp	78	15,349 bp	263 bp
18985	NMB.CR026.3B	*Chelonoidis alburyorum*	Crooked Island (1702 Cave)	2740-2490	65 mg	12.50 ng/µl	12.5 ng	22,982,414	6,637,046	21,046	LT599482	78 bp	110	15,349 bp	1,742 bp
18987	GT-3, FS 234	*Chelonoidis alburyorum*	Grand Turk (Coralie)	1179-1047, 1032-985	51 mg	11.00 ng/µl	13.2 ng	4,015,772	1,581,177	97,211	LT599482	76 bp	482	15,349 bp	0 bp
18988	MC-37, Unit 4, II/7	*Chelonoidis alburyorum*	Middle Caicos (Indian Cave)	1300-1240, 1200-1180	56 mg	2.86 ng/µl	13.2 ng	10,201,099	4,108,535	13,869	LT599482	71 bp	67	15,349 bp	1,307 bp
18989	NMB.MY014.3	*Chelonoidis alburyorum*	Mayaguana (Abraham's Bay Cave)	n/a	51 mg	1.63 ng/µl	13.0 ng	11,797,303	2,762,525	1,355	LT599482	72 bp	9	15,329 bp	2,663 bp
18990	NMB.CR05	*Chelonoidis alburyorum*	Crooked Island (McKay's Bluff Cave)	925-785	60 mg	11.10 ng/µl	13.3 ng	11,712,201	3,403,619	8,967	LT599482	74 bp	12	15,288 bp	1,097 bp
18991	NMB.EL180.27	*Chelonoidis alburyorum*	Eleuthera (Kelly's Blue Hole)	n/a	56 mg	0.96 ng/µl	13.0 ng	13,244,464	5,388,839	1,875	LT599482	74 bp	12	15,335 bp	1,740 bp
18994	NMB.AB52.4	*Chelonoidis alburyorum*	Great Abaco (Lost Reel Cave)	1230-1210, 1180-1060	61 mg	2.42 ng/µl	13.1 ng	10,856,727	5,956,335	1,710	LT599482	72 bp	11	15,346 bp	1,586 bp
	**Samples that produced poor sequence data**														
18986	NMB.LI108	*Chelonoidis alburyorum*	Long Island (Hanging Garden Crawl Through)	n/a	55 mg	2.46 ng/µl	13.0 ng	2,609,964	905,876	342	LT599482	n/a	5	The assembled reads turned out to be contaminations when being submitted to a BLAST search.	
18978	CTES-PZ 7781-2	*Chelonoidis* cf. *lutzae*	Argentina (Corrientes, Bella Vista)	58-22 Ka	60 mg	too low	<0.2 ng	3,626,488	290,823	252	LT599484	n/a	4		
18979	CTES-PZ 7781-1	*Chelonoidis* cf. *lutzae*	Argentina (Corrientes, Bella Vista)	58-22 Ka	59 mg	0.18 ng/µl	3.6 ng	3,187,688	872,955	643	LT599484	n/a	7		
18980	CTES-PZ 7391*	*Chelonoidis lutzae*	Argentina (Corrientes, Bella Vista)	58-22 Ka	61 mg	too low	<0.2 ng	3,601,652	1,334,305	1,111	LT599484	n/a	9		
18981	CTES-PZ 7101	*Chelonoidis* cf. *lutzae*	Argentina (Corrientes, Bella Vista)	58-22 Ka	58 mg	0.21 ng/µl	4.2 ng	2,829,342	519,908	366	LT599484	n/a	5		
		Chelonoidis spec.	Venezuela (Estado Falcón, Taratara)	n/a	64 mg	6.86 ng/µl	13.7 ng	7,095,151	3,050,300	2,819	LT599484	n/a	18		
	MHD 1000*	*Chelonoidis dominicensis*	Dominican Republic (Oleg's Bat Cave)	8644–8538***	46 mg	1.92 ng/µl	13.4 ng	7,460,584	4,113,903	179	LT599482	n/a	4		
	**Blanks**														
		EB1						1,855,944	306,697	26	LT599482	n/a	3		
		EB2						2,152,519	186,130	193	LT599482	n/a	4		
		LB						2,156,486	54,974	280	LT599482	n/a	4		

* Holotype.

** See Table 1 for Bahamian material.

*** ^{14}C age (cal BP), conventional age 7810 ± 30 BP. Previously unpublished data; lab analyses were the same as those in Steadman *et al*. (2020).

Table S2. Example of a contamination screening using FastQScreen for sample NMB.MY014.3 to assess endogenous DNA content in relation to potential contamination sources. Reported are numbers of reads that map to a given set of reference mt-genomes. In a first step, identified non-target reads, i.e., reads that uniquely mapped to the non-tortoise mt-genomes, were excluded, resulting in readpool 1. Then, reads uniquely mapping to the reference of *Chelonoidis alburyorum* were copied into a new file (readpool 2). Both readpools were used for subsequent mt-genome assembly (see main text).

File	Reads processed	Unmapped	One hit / one genome	Multiple hits / one genome	One hit / multiple genomes	Multiple hits / multiple genomes
Homo	2,762,525	2,733,054	15,163	2	14,306	0
Penicillium	2,762,525	2,762,510	11	4	0	0
Bacillus	2,762,525	2,759,152	92	2,025	3	1,253
Ecoli	2,762,525	2,759,874	119	1,275	2	1,255
Felis	2,762,525	2,751,803	145	0	10,577	0
Ursus	2,762,525	2,751,648	105	0	10,772	0
Gulo	2,762,525	2,749,818	76	157	12,474	0
Canis	2,762,525	2,750,452	135	0	11,938	0
Sus	2,762,525	2,751,922	288	0	10,315	0
Bos	2,762,525	2,752,674	43	0	9,808	0
Gallus	2,762,525	2,751,564	4,391	0	6,570	0
Sula	2,762,525	2,755,792	162	0	6,571	0
Mus	2,762,525	2,752,050	73	0	10,402	0
Cyprinus	2,762,525	2,755,182	18	0	7,325	0
Hyles	2,762,525	2,762,524	1	0	0	0
Chelonoidis alburyorum	2,762,525	2,756,416	1,492	1	4,616	0

Table S3. The best evolutionary models and partitioning schemes as determined by PartitionFinder2 applying the greedy search scheme and the Bayesian Information Criterion.

**RAxML**		
**Subset**	**Best Model**	**Partition names**
1	GTR+G	Block13_tRNA, Block24_ND6_pos1, Block1_tRNA
2	GTR+I+G	Block4_16S, Block22_tRNA, Block2_12S, Block3_tRNA, Block19_tRNA, Block27_tRNA
3	GTR+I+G	Block5_tRNA, Block26_cytb_pos1, Block9_tRNA, Block25_tRNA, Block7_tRNA, Block11_tRNA, Block17_tRNA
4	GTR+I+G	Block18_ND3_pos1, Block6_ND1_pos1, Block20_ND4L_pos1, Block8_ND2_pos1, Block14_atp8_pos2, Block21_ND4_pos1, Block15_atp6_pos1, Block23_ND5_pos1, Block14_atp8_pos1, Block24_ND6_pos3
5	GTR+I+G	Block8_ND2_pos2, Block18_ND3_pos2, Block20_ND4L_pos2, Block23_ND5_pos2, Block26_cytb_pos2, Block15_atp6_pos2, Block21_ND4_pos2, Block6_ND1_pos2, Block16_coxIII_pos2, Block12_coxII_pos2
6	GTR+I+G	Block15_atp6_pos3, Block21_ND4_pos3, Block23_ND5_pos3, Block20_ND4L_pos3, Block26_cytb_pos3, Block6_ND1_pos3, Block24_ND6_pos2, Block8_ND2_pos3
7	GTR+I+G	Block10_coxI_pos1, Block16_coxIII_pos1, Block12_coxII_pos1
8	GTR+I+G	Block10_coxI_pos2
9	GTR+I+G	Block16_coxIII_pos3, Block14_atp8_pos3, Block12_coxII_pos3, Block10_coxI_pos3, Block18_ND3_pos3
**MrBayes**		
**Subset**	**Best Model**	**Partition names**
1	GTR+I+G	Block20_ND4L_pos1, Block14_atp8_pos2, Block18_ND3_pos1, Block6_ND1_pos1, Block8_ND2_pos1, Block21_ND4_pos1, Block15_atp6_pos1, Block14_atp8_pos1, Block27_tRNA, Block24_ND6_pos3, Block23_ND5_pos1, Block1_tRNA
2	GTR+I+G	Block2_12S, Block4_16S
3	GTR+I+G	Block22_tRNA, Block3_tRNA, Block25_tRNA, Block19_tRNA, Block7_tRNA, Block5_tRNA, Block9_tRNA, Block26_cytb_pos1, Block11_tRNA, Block17_tRNA
4	GTR+I+G	Block12_coxII_pos2, Block16_coxIII_pos2, Block6_ND1_pos2, Block26_cytb_pos2, Block15_atp6_pos2, Block21_ND4_pos2
5	GTR+I+G	Block16_coxIII_pos3, Block15_atp6_pos3, Block21_ND4_pos3, Block20_ND4L_pos3, Block23_ND5_pos3, Block8_ND2_pos3, Block6_ND1_pos3, Block26_cytb_pos3, Block24_ND6_pos2
6	HKY+I+G	Block8_ND2_pos2, Block18_ND3_pos2, Block20_ND4L_pos2, Block23_ND5_pos2
7	SYM+I+G	Block12_coxII_pos1, Block16_coxIII_pos1, Block10_coxI_pos1
8	HKY+I	Block10_coxI_pos2
9	GTR+I+G	Block18_ND3_pos3, Block14_atp8_pos3, Block10_coxI_pos3, Block12_coxII_pos3
10	HKY+I+G	Block13_tRNA, Block24_ND6_pos1

Table S4. Data blocks of the alignment used for phylogenetic analyses.

Block1_tRNA	= 1–26;
Block2_12S	= 27–1040;
Block3_tRNA	= 1041–1115;
Block4_16S	= 1116–2803;
Block5_tRNA	= 2804–2880;
Block6_ND1_pos1	= 2881–3858\3;
Block6_ND1_pos2	= 2882–3858\3;
Block6_ND1_pos3	= 2883–3858\3;
Block7_tRNA	= 3859–4070;
Block8_ND2_pos1	= 4071–5108\3;
Block8_ND2_pos2	= 4072–5108\3;
Block8_ND2_pos3	= 4073–5108\3;
Block9_tRNA	= 5109–5490;
Block10_coxI_pos1	= 5491–7029\3;
Block10_coxI_pos2	= 5492–7029\3;
Block10_coxI_pos3	= 5493–7029\3;
Block11_tRNA	= 7030–7164;
Block12_coxII_pos1	= 7165–7851\3;
Block12_coxII_pos2	= 7166–7851\3;
Block12_coxII_pos3	= 7167–7851\3;
Block13_tRNA	= 7852–7929;
Block14_atp8_pos1	= 7930–8100\3;
Block14_atp8_pos2	= 7931–8100\3;
Block14_atp8_pos3	= 7932–8100\3;
Block15_atp6_pos1	= 8101–8769\3;
Block15_atp6_pos2	= 8102–8769\3;
Block15_atp6_pos3	= 8103–8769\3;
Block16_coxIII_pos1	= 8770–9549\3;
Block16_coxIII_pos2	= 8771–9549\3;
Block16_coxIII_pos3	= 8772–9549\3;
Block17_tRNA	= 9550–9619;
Block18_ND3_pos1	= 9620–9967\3;
Block18_ND3_pos2	= 9621–9967\3;
Block18_ND3_pos3	= 9622–9967\3;
Block19_tRNA	= 9968–10040;
Block20_ND4L_pos1	= 10041–10331\3;
Block20_ND4L_pos2	= 10042–10331\3;
Block20_ND4L_pos3	= 10043–10331\3;
Block21_ND4_pos1	= 10332–11705\3;
Block21_ND4_pos2	= 10333–11705\3;
Block21_ND4_pos3	= 10334–11705\3;
Block22_tRNA	= 11706–11920;
Block23_ND5_pos1	= 11921–13738\3;
Block23_ND5_pos2	= 11922–13738\3;
Block23_ND5_pos3	= 11923–13738\3;
Block24_ND6_pos3	= 13739–14273\3;
Block24_ND6_pos2	= 13740–14273\3;
Block24_ND6_pos1	= 13741–14273\3;
Block25_tRNA	= 14274–14343;
Block26_cytb_pos1	= 14344–15486\3;
Block26_cytb_pos2	= 14345–15486\3;
Block26_cytb_pos3	= 14346–15486\3;
Block27_tRNA	= 15487–15516;

Table S5. Nomenclatural history of *Chelonoidis alburyorum* specimens with aDNA sequences reported in this paper. The vertical sequence here matches the vertical sequence in Figure 3. Specimens are in the collection of the National Museum of The Bahamas (NMB), except for Grand Turk and Middle Caicos, which are in the Turks and Caicos National Museum and for which we provide field provenience. See Tables 1 and S1 for further information.

**Island, site**	**Specimen**	**Accession number**	**Franz & Franz (2009)**	**Kehlmaier *et al*. (2017)**	**Steadman *et al*. (2020)**	**Franz *et al*. (2020)**
Crooked Island, 1702 Cave	NMB.CR026.3B	LR968546	—	—	*Chelonoidis* new sp. C	—
Great Abaco, Sawmill Sink	NMB.AB50.0008	LT599482	*C. alburyorum* new species	*C. alburyorum*	*C. alburyorum*	*C. a. alburyorum*
Crooked Island, 1702 Cave	NMB.CR026.3A	LR968545	—	—	*Chelonoidis* new sp. C	—
Eleuthera, Kelly's Blue Hole	NMB.EL180.27	LR968551	*Chelonoidis* sp.	—	*Chelonoidis* new sp. B	—
Mayaguana, Abraham's Bay Cave	NMB.MY014.3	LR968549	*Chelonoidis* sp.	—	*Chelonoidis* new sp. D	—
Great Abaco, Lost Reel Cave	NMB.AB52.4	LR968552	*Chelonoidis* sp.	—	*Chelonoidis* new sp. A	—
Grand Turk, Coralie	GT-3, FS 82	LR968543	*Chelonoidis* sp.	—	*Chelonoidis* new sp. F	*C. a. keegani* new subspecies
Grand Turk, Coralie	GT-3, FS 234	LR968547	*Chelonoidis* sp.	—	*Chelonoidis* new sp. F	*C. a. keegani* new subspecies
Middle Caicos, Indian Cave	MC-37, Unit 9, surface	LR968544	*Chelonoidis* sp.	—	*Chelonoidis* new sp. E	*C. a. sementis* new subspecies
Middle Caicos, Indian Cave	MC-37, Unit 4, II/7	LR968548	*Chelonoidis* sp.	—	*Chelonoidis* new sp. E	*C. a. sementis* new subspecies
Crooked Island, McKay's Bluff Cave	NMB.CR05	LR968550	—	—	*Chelonoidis* new sp. C	—

Table S6. Uncorrected *p* distances (percentages) of 66 mitogenome sequences (15,516 positions; tortoise dataset and outgroups), calculated in MEGA X using the pairwise deletion option. Codes preceding taxon names are ENA/GenBank accession numbers.

		1	2	3	4	5	6	7	8	9	10	11	12	13	14	15	16	17	18	19	20	21	22	23	24	25	26	27	28	29	30	31	32	33	34	35	36	37	38	39	40	41	42	43	44	45	46	47	48	49	50	51	52	53	54	55	56	57	58	59	60	61	62	63	64	65	66
1	AF069423 *Chrysemys picta*	—
2	FJ469674 *Mauremys reevesii*	17.9%	—
3	LR697067 *Aldabrachelys gigantea*	17.1%	14.6%	—
4	LR697068 *Astrochelys radiata*	17.5%	14.9%	8.8%	—
5	LR697069 *Astrochelys yniphora*	17.7%	15.2%	9.5%	8.8%	—
6	LT599484 *Chelonoidis chilensis*	17.7%	15.2%	10.6%	10.9%	11.5%	—
7	LT599483 *Chelonoidis carbonarius*	17.7%	15.2%	10.8%	11.4%	11.8%	10.4%	—
8	MT017704 *Chelonoidis carbonarius*	17.7%	15.3%	11.0%	11.7%	12.1%	10.8%	1.7%	—
9	LT599485 *Chelonoidis denticulatus*	17.8%	15.4%	10.9%	11.1%	11.8%	10.5%	9.5%	9.8%	—
10	MT017703 *Chelonoidis denticulatus*	17.8%	15.5%	10.8%	11.0%	11.6%	10.6%	9.6%	9.8%	0.2%	—
11	LT599486 *Chelonoidis vicina*	17.1%	14.7%	9.7%	10.2%	10.7%	8.5%	9.5%	9.9%	9.4%	9.4%	—
12	MG912820 *Chelonoidis duncanensis*	17.0%	14.7%	9.7%	10.2%	10.6%	8.5%	9.5%	9.7%	9.3%	9.3%	0.6%	—
13	MT017702 *Chelonoidis microphyes*	17.1%	14.7%	9.8%	10.3%	10.6%	8.5%	9.6%	9.9%	9.5%	9.4%	0.1%	0.6%	—
14	MT017701 *Chelonoidis vandenburghi*	17.1%	14.7%	9.8%	10.3%	10.6%	8.5%	9.6%	9.9%	9.5%	9.4%	0.1%	0.6%	0.1%	—
15	MT017700 *Chelonoidis guntheri*	17.1%	14.8%	9.8%	10.2%	10.6%	8.6%	9.6%	9.9%	9.4%	9.4%	0.1%	0.7%	0.2%	0.2%	—
16	MT017699 *Chelonoidis vicina*	17.1%	14.7%	9.7%	10.2%	10.6%	8.5%	9.6%	9.9%	9.4%	9.3%	0.1%	0.7%	0.2%	0.2%	0.1%	—
17	MT017698 *Chelonoidis porteri*	17.1%	14.7%	9.7%	10.2%	10.6%	8.5%	9.6%	9.9%	9.4%	9.3%	0.3%	0.6%	0.3%	0.3%	0.3%	0.3%	—
18	MT017697 *Chelonoidis duncanensis*	17.0%	14.7%	9.7%	10.2%	10.5%	8.4%	9.5%	9.8%	9.4%	9.3%	0.6%	0.0%	0.7%	0.7%	0.7%	0.7%	0.7%	—
19	MT017696 *Chelonoidis hoodensis*	17.1%	14.7%	9.7%	10.2%	10.6%	8.5%	9.6%	9.9%	9.4%	9.4%	0.8%	0.8%	0.8%	0.8%	0.8%	0.8%	0.8%	0.8%	—
20	MT017695 *Chelonoidis donfaustoi*	17.1%	14.8%	9.7%	10.3%	10.6%	8.4%	9.6%	9.9%	9.5%	9.4%	0.8%	0.8%	0.8%	0.8%	0.8%	0.8%	0.8%	0.8%	0.4%	—
21	MT017694 *Chelonoidis darwini*	17.2%	14.8%	9.6%	10.3%	10.5%	8.4%	9.5%	9.8%	9.4%	9.4%	**0.9%**	0.8%	**0.9%**	**0.9%**	**0.9%**	**0.9%**	**0.9%**	0.8%	0.6%	0.6%	—
22	MT017693 *Chelonoidis becki*	17.2%	14.8%	9.6%	10.3%	10.6%	8.4%	9.5%	9.8%	9.4%	9.4%	**0.9%**	**0.9%**	**0.9%**	**0.9%**	**0.9%**	**0.9%**	**0.9%**	**0.9%**	0.6%	0.7%	0.0%	—
23	MT017690 *Chelonoidis abingdonii*	17.0%	14.6%	9.7%	10.2%	10.6%	8.4%	9.4%	9.8%	9.4%	9.3%	0.8%	0.7%	0.8%	0.8%	0.8%	0.8%	0.7%	0.8%	0.2%	0.4%	0.6%	0.6%	—
24	MT017691 *Chelonoidis niger*	17.0%	14.6%	9.6%	9.9%	10.4%	8.4%	9.4%	9.6%	9.3%	9.3%	0.3%	0.6%	0.3%	0.3%	0.3%	0.3%	0.3%	0.6%	0.7%	0.8%	0.8%	0.8%	0.7%	—
25	MT017692 *Chelonoidis chathamensis*	17.1%	14.7%	9.7%	10.2%	10.5%	8.4%	9.5%	9.8%	9.4%	9.3%	0.8%	0.7%	0.8%	0.8%	0.8%	0.8%	0.7%	0.8%	0.4%	0.2%	0.5%	0.6%	0.3%	0.7%	—
26	LT599482 *Chelonoidis alburyorum* GREAT ABACO	17.3%	14.9%	9.9%	10.2%	10.7%	9.2%	9.7%	10.2%	9.5%	9.5%	7.6%	7.6%	7.7%	7.7%	7.7%	7.7%	7.7%	7.6%	7.6%	7.6%	7.5%	7.6%	7.5%	7.6%	7.6%	—
27	LR968545 *Chelonoidis alburyorum* CROOKED ISLAND	17.3%	15.0%	9.9%	10.2%	10.6%	9.2%	9.8%	10.2%	9.5%	9.5%	7.6%	7.5%	7.7%	7.7%	7.6%	7.6%	7.6%	7.6%	7.6%	7.6%	7.5%	7.5%	7.4%	7.5%	7.5%	0.1%	—
28	LR968546 *Chelonoidis alburyorum* CROOKED ISLAND	17.1%	14.7%	9.7%	9.8%	10.4%	9.0%	9.7%	10.1%	9.4%	9.3%	7.3%	7.3%	7.4%	7.4%	7.4%	7.4%	7.4%	7.4%	7.4%	7.4%	7.3%	7.3%	7.2%	7.3%	7.3%	0.2%	0.1%	—
29	LR968544 *Chelonoidis alburyorum* MIDDLE CAICOS	17.2%	14.8%	9.9%	10.2%	10.6%	9.2%	9.7%	10.1%	9.4%	9.4%	7.6%	7.5%	7.6%	7.6%	7.6%	7.6%	7.6%	7.5%	7.6%	7.6%	7.5%	7.5%	7.4%	7.5%	7.6%	**0.7%**	**0.7%**	0.5%	—
30	LR968547 *Chelonoidis alburyorum* GRAND TURK	17.2%	14.8%	9.9%	10.2%	10.5%	9.2%	9.7%	10.1%	9.4%	9.4%	7.5%	7.5%	7.6%	7.6%	7.6%	7.6%	7.6%	7.5%	7.5%	7.6%	7.5%	7.5%	7.4%	7.5%	7.5%	**0.7%**	**0.7%**	0.5%	0.3%	—
31	LR968543 *Chelonoidis alburyorum* GRAND TURK	17.2%	14.8%	9.9%	10.2%	10.5%	9.2%	9.7%	10.1%	9.4%	9.4%	7.5%	7.4%	7.6%	7.6%	7.6%	7.6%	7.5%	7.5%	7.5%	7.5%	7.5%	7.5%	7.3%	7.5%	7.5%	0.6%	0.6%	0.4%	0.3%	0.0%	—
32	LR968548 *Chelonoidis alburyorum* MIDDLE CAICOS	16.8%	14.4%	9.5%	10.0%	10.2%	8.8%	9.5%	9.9%	9.1%	9.1%	7.2%	7.2%	7.3%	7.3%	7.2%	7.2%	7.2%	7.2%	7.2%	7.2%	7.2%	7.2%	7.0%	7.1%	7.2%	0.6%	0.6%	0.4%	0.2%	0.1%	0.1%	—
33	LR968549 *Chelonoidis alburyorum* MAYAGUANA	16.5%	14.0%	9.4%	9.7%	10.1%	8.5%	9.1%	9.6%	8.9%	9.0%	6.9%	6.9%	7.0%	7.0%	6.9%	6.9%	6.9%	6.9%	6.9%	7.0%	6.9%	6.9%	6.8%	6.9%	6.9%	0.6%	0.5%	0.3%	0.2%	0.1%	0.1%	0.1%	—
34	LR968550 *Chelonoidis alburyorum* CROOKED ISLAND	16.5%	14.2%	9.7%	9.9%	10.3%	8.6%	9.3%	9.8%	9.1%	9.1%	7.0%	7.0%	7.1%	7.1%	7.0%	7.1%	7.0%	7.0%	7.0%	7.0%	6.9%	6.9%	6.8%	7.0%	7.0%	0.5%	0.5%	0.3%	0.2%	0.1%	0.1%	0.1%	0.1%	—
35	LR968551 *Chelonoidis alburyorum* ELEUTHERA	16.9%	14.5%	9.6%	9.8%	10.3%	8.9%	9.4%	9.9%	9.2%	9.2%	7.2%	7.1%	7.3%	7.3%	7.2%	7.2%	7.2%	7.2%	7.2%	7.3%	7.2%	7.2%	7.0%	7.1%	7.2%	0.5%	0.5%	0.3%	0.2%	0.1%	0.1%	0.1%	0.0%	0.0%	—
36	LR968552 *Chelonoidis alburyorum* GREAT ABACO	16.9%	14.3%	9.5%	9.9%	10.3%	8.7%	9.3%	9.6%	9.0%	9.0%	7.0%	6.9%	7.1%	7.1%	7.1%	7.1%	7.0%	7.0%	7.0%	7.0%	6.9%	7.0%	6.8%	6.9%	7.0%	0.6%	0.5%	0.4%	0.2%	0.1%	0.1%	0.1%	0.0%	0.1%	0.0%	—
37	LR697065 *Cylindraspis triserrata*	17.6%	14.6%	10.5%	11.2%	11.6%	11.4%	11.4%	11.6%	11.8%	11.8%	10.5%	10.4%	10.5%	10.5%	10.5%	10.5%	10.5%	10.4%	10.4%	10.4%	10.4%	10.4%	10.4%	10.3%	10.4%	10.8%	10.8%	10.6%	10.7%	10.7%	10.7%	10.4%	10.0%	10.2%	10.4%	10.3%	—
38	LR697066 *Cylindraspis vosmaeri*	17.4%	14.5%	11.0%	11.2%	11.8%	11.7%	11.8%	12.0%	11.7%	11.9%	10.8%	10.9%	10.9%	10.9%	10.9%	10.9%	10.8%	10.9%	10.8%	10.9%	10.8%	10.9%	10.8%	10.9%	10.8%	11.0%	11.0%	10.8%	10.9%	10.9%	10.9%	10.5%	10.3%	10.5%	10.5%	10.4%	9.1%	—
39	LR697060 *Cylindraspis indica*	17.5%	14.9%	11.0%	11.5%	11.7%	11.5%	12.0%	12.2%	11.9%	12.0%	10.8%	10.8%	10.9%	10.9%	10.8%	10.8%	10.7%	10.8%	10.8%	10.7%	10.8%	10.8%	10.7%	10.8%	10.7%	10.9%	10.9%	10.6%	10.8%	10.8%	10.9%	10.5%	10.4%	10.5%	10.6%	10.4%	9.1%	5.9%	—
40	LR697062 *Cylindraspis inepta*	17.5%	14.9%	10.9%	11.4%	11.6%	11.4%	11.8%	12.0%	11.7%	11.8%	10.6%	10.6%	10.6%	10.6%	10.6%	10.6%	10.5%	10.6%	10.6%	10.6%	10.6%	10.6%	10.5%	10.5%	10.6%	10.8%	10.7%	10.5%	10.7%	10.7%	10.7%	10.4%	10.2%	10.3%	10.4%	10.2%	9.2%	5.9%	1.8%	—
41	LR697064 *Cylindraspis peltastes*	17.5%	14.6%	11.1%	11.4%	11.9%	11.9%	11.9%	12.0%	11.8%	11.9%	11.0%	11.1%	11.1%	11.1%	11.1%	11.1%	11.0%	11.1%	11.0%	11.0%	11.0%	11.0%	11.0%	11.0%	11.0%	11.2%	11.2%	11.0%	11.2%	11.2%	11.2%	10.8%	10.6%	10.9%	10.8%	10.7%	9.2%	1.9%	6.2%	6.2%	—
42	LR697070 *Chersina angulata*	17.4%	15.4%	10.8%	11.1%	11.4%	11.6%	12.1%	12.3%	11.9%	11.9%	11.1%	10.9%	11.2%	11.2%	11.2%	11.1%	11.1%	11.0%	11.1%	11.2%	11.0%	11.1%	11.0%	10.9%	11.0%	11.0%	11.0%	10.6%	11.0%	11.0%	11.0%	10.8%	10.4%	10.7%	10.6%	10.6%	11.7%	11.8%	11.8%	11.6%	11.9%	—
43	LR697072 *Geochelone elegans*	17.9%	15.7%	11.3%	12.1%	12.3%	11.6%	11.8%	12.1%	11.9%	11.9%	10.9%	10.9%	11.0%	11.0%	11.0%	11.0%	11.0%	10.9%	10.9%	10.9%	10.9%	10.9%	10.9%	10.9%	10.9%	11.0%	11.0%	10.7%	11.0%	11.0%	11.0%	10.6%	10.2%	10.5%	10.7%	10.6%	12.5%	12.5%	12.2%	12.2%	12.6%	12.4%	—
44	LR697073 *Geochelone platynota*	18.2%	15.7%	11.6%	12.2%	12.4%	11.7%	11.9%	12.2%	12.0%	12.1%	10.9%	10.9%	11.0%	11.0%	11.0%	11.0%	10.9%	10.9%	10.9%	10.9%	10.9%	10.9%	10.9%	10.9%	10.8%	10.9%	10.9%	10.6%	11.0%	11.0%	10.9%	10.5%	10.1%	10.4%	10.6%	10.4%	12.7%	12.6%	12.5%	12.4%	12.7%	12.5%	3.2%	—
45	LT599487 *Centrochelys sulcata*	17.5%	15.5%	10.7%	11.4%	11.7%	10.8%	11.1%	11.4%	10.7%	10.8%	9.8%	9.8%	9.9%	9.9%	9.9%	9.9%	9.8%	9.8%	9.8%	9.8%	9.8%	9.8%	9.8%	9.8%	9.8%	10.0%	10.0%	9.9%	10.0%	9.9%	9.9%	9.6%	9.3%	9.5%	9.6%	9.5%	11.7%	11.8%	11.8%	11.6%	11.8%	11.9%	9.7%	9.6%	—
46	LR697074 *Gopherus berlandieri*	17.4%	15.3%	13.3%	13.7%	13.8%	14.2%	14.4%	14.6%	14.3%	14.2%	13.5%	13.4%	13.5%	13.5%	13.5%	13.5%	13.5%	13.4%	13.5%	13.5%	13.5%	13.5%	13.4%	13.4%	13.5%	13.4%	13.4%	13.1%	13.3%	13.3%	13.3%	12.9%	12.6%	12.8%	12.9%	12.8%	13.8%	13.7%	13.7%	13.8%	13.9%	14.1%	14.7%	14.7%	13.8%	—
47	LR697075 *Homopus areolatus*	18.2%	15.7%	11.6%	11.6%	12.3%	12.4%	12.7%	13.0%	12.5%	12.5%	11.6%	11.6%	11.7%	11.7%	11.6%	11.6%	11.6%	11.6%	11.6%	11.7%	11.6%	11.6%	11.6%	11.5%	11.6%	11.9%	11.9%	11.6%	11.9%	11.9%	11.9%	11.6%	11.4%	11.5%	11.5%	11.5%	12.1%	12.4%	12.6%	12.5%	12.6%	11.3%	13.5%	13.5%	12.6%	14.4%	—
48	LR697071 *Chersobius boulengeri*	18.6%	16.4%	12.5%	12.7%	13.1%	13.2%	13.5%	13.8%	13.5%	13.6%	12.6%	12.5%	12.6%	12.6%	12.6%	12.6%	12.5%	12.5%	12.6%	12.6%	12.6%	12.6%	12.5%	12.5%	12.5%	12.4%	12.5%	12.2%	12.4%	12.5%	12.4%	12.1%	11.9%	12.1%	12.2%	12.0%	12.9%	13.1%	13.3%	13.2%	13.3%	9.9%	14.4%	14.2%	13.6%	15.3%	12.8%	—
49	DQ080043 *Indotestudo elongata*	17.7%	15.2%	12.1%	12.5%	12.7%	12.7%	12.7%	12.7%	12.8%	12.7%	11.9%	11.8%	11.9%	11.9%	11.9%	11.9%	11.8%	11.8%	11.9%	11.9%	11.8%	11.8%	11.8%	11.7%	11.8%	11.9%	11.9%	11.5%	11.8%	11.8%	11.8%	11.4%	11.3%	11.4%	11.5%	11.4%	12.3%	12.5%	12.5%	12.3%	12.7%	12.7%	13.7%	13.7%	12.6%	14.0%	13.3%	14.0%	—
50	DQ080044 *Indotestudo forstenii*	17.9%	15.3%	12.2%	12.8%	12.8%	13.0%	13.0%	13.1%	13.2%	13.1%	12.1%	12.1%	12.2%	12.2%	12.2%	12.2%	12.1%	12.1%	12.2%	12.1%	12.1%	12.1%	12.1%	12.0%	12.1%	12.3%	12.3%	12.0%	12.2%	12.3%	12.2%	11.8%	11.7%	11.8%	12.0%	11.9%	12.4%	12.5%	12.6%	12.4%	12.6%	12.8%	13.8%	13.8%	12.9%	14.2%	13.6%	14.0%	4.5%	—
51	LR697076 *Kinixys erosa*	18.5%	16.1%	12.1%	12.7%	12.8%	12.3%	12.4%	12.6%	12.3%	12.2%	11.7%	11.6%	11.7%	11.7%	11.7%	11.7%	11.7%	11.6%	11.6%	11.6%	11.6%	11.6%	11.5%	11.5%	11.5%	11.8%	11.8%	11.4%	11.8%	11.8%	11.7%	11.3%	11.1%	11.2%	11.3%	11.2%	13.0%	12.9%	12.9%	12.9%	13.0%	12.7%	13.3%	13.4%	12.6%	15.1%	13.3%	14.0%	13.9%	14.1%	—
52	LR697077 *Kinixys spekii*	17.9%	15.1%	11.3%	11.8%	11.8%	11.5%	11.8%	11.9%	11.4%	11.3%	10.4%	10.4%	10.4%	10.4%	10.4%	10.4%	10.5%	10.4%	10.4%	10.5%	10.4%	10.4%	10.4%	10.3%	10.4%	10.6%	10.6%	10.4%	10.6%	10.6%	10.6%	10.2%	9.9%	10.2%	10.3%	10.2%	11.8%	12.0%	12.2%	12.0%	12.1%	11.9%	12.5%	12.5%	11.7%	14.2%	12.4%	13.2%	12.8%	13.2%	8.0%	—
53	DQ080042 *Malacochersus tornieri*	18.5%	16.5%	13.5%	14.2%	14.2%	14.1%	14.2%	14.4%	14.3%	14.4%	13.3%	13.3%	13.4%	13.4%	13.4%	13.3%	13.3%	13.3%	13.3%	13.4%	13.2%	13.3%	13.3%	13.3%	13.3%	13.2%	13.2%	13.1%	13.1%	13.2%	13.1%	12.8%	12.4%	12.8%	12.9%	12.7%	13.5%	13.5%	13.7%	13.6%	13.7%	13.9%	14.7%	14.7%	13.8%	15.3%	14.4%	15.2%	11.3%	11.4%	15.2%	14.2%	—
54	DQ080040 *Manouria emys*	17.2%	14.1%	12.7%	13.4%	13.5%	13.5%	13.6%	13.8%	13.6%	13.4%	12.6%	12.4%	12.6%	12.6%	12.6%	12.5%	12.5%	12.3%	12.5%	12.5%	12.4%	12.5%	12.5%	12.4%	12.6%	12.9%	12.8%	12.7%	12.8%	12.8%	12.8%	12.6%	12.2%	12.5%	12.6%	12.4%	13.2%	13.2%	13.3%	13.2%	13.3%	13.7%	14.5%	14.4%	13.4%	12.9%	14.1%	14.8%	13.4%	13.8%	14.5%	13.7%	14.7%	—
55	EF661586 *Manouria impressa*	17.4%	14.3%	13.1%	13.4%	13.3%	13.9%	13.7%	13.9%	13.8%	13.7%	13.1%	13.0%	13.1%	13.1%	13.1%	13.1%	13.0%	13.0%	13.0%	13.1%	13.0%	13.0%	13.0%	12.9%	13.1%	13.2%	13.2%	12.9%	13.1%	13.1%	13.1%	12.9%	12.7%	12.8%	12.9%	12.8%	13.4%	13.4%	13.5%	13.4%	13.5%	13.8%	14.5%	14.5%	13.4%	13.2%	14.3%	14.9%	13.2%	13.5%	14.9%	13.8%	14.5%	8.7%	—
56	LR697078 *Psammobates geometricus*	18.2%	15.7%	11.6%	11.9%	12.5%	12.1%	12.6%	12.9%	12.5%	12.5%	11.7%	11.6%	11.8%	11.8%	11.8%	11.8%	11.7%	11.7%	11.7%	11.8%	11.7%	11.7%	11.6%	11.7%	11.7%	11.5%	11.4%	11.3%	11.4%	11.4%	11.4%	11.2%	10.9%	11.0%	11.2%	11.1%	12.1%	12.1%	12.4%	12.4%	12.3%	11.7%	13.4%	13.5%	12.4%	14.6%	12.0%	12.8%	13.4%	13.4%	13.3%	12.6%	14.5%	13.8%	14.2%	—
57	LR697079 *Psammobates oculifer*	19.5%	17.3%	13.7%	13.9%	14.4%	14.0%	14.5%	14.3%	14.4%	14.3%	13.7%	13.6%	13.7%	13.8%	13.7%	13.7%	13.7%	13.6%	13.7%	13.7%	13.6%	13.6%	13.6%	13.5%	13.7%	13.6%	13.6%	13.4%	13.6%	13.6%	13.6%	13.4%	13.2%	13.3%	13.5%	13.3%	14.2%	14.1%	14.1%	14.0%	14.2%	13.5%	15.2%	15.2%	14.2%	16.0%	13.6%	14.3%	14.9%	15.2%	14.9%	14.3%	15.9%	15.7%	16.0%	10.2%	—
58	LR697080 *Pyxis arachnoides*	18.0%	15.8%	10.9%	10.9%	11.7%	12.5%	12.8%	12.9%	12.8%	13.0%	12.3%	12.3%	12.3%	12.3%	12.3%	12.3%	12.2%	12.2%	12.2%	12.2%	12.2%	12.2%	12.1%	12.1%	12.2%	12.3%	12.3%	12.1%	12.4%	12.4%	12.4%	12.1%	11.7%	11.9%	12.1%	11.9%	12.7%	13.1%	13.2%	13.0%	13.1%	12.5%	13.5%	13.7%	13.0%	14.9%	13.3%	13.9%	13.7%	13.9%	13.5%	12.9%	14.7%	14.5%	14.7%	13.3%	14.9%	—
59	LR697081 *Pyxis planicauda*	18.3%	16.4%	11.6%	11.3%	12.1%	13.0%	12.9%	13.2%	13.1%	13.1%	12.5%	12.5%	12.5%	12.5%	12.5%	12.5%	12.5%	12.4%	12.4%	12.5%	12.4%	12.5%	12.4%	12.3%	12.4%	12.6%	12.6%	12.4%	12.7%	12.6%	12.6%	12.4%	12.0%	12.2%	12.4%	12.3%	13.1%	13.5%	13.8%	13.7%	13.4%	12.8%	14.3%	14.2%	13.5%	15.1%	13.6%	13.8%	14.2%	14.4%	13.9%	13.1%	15.2%	14.9%	15.0%	13.7%	15.3%	7.1%	—
60	LR697082 *Stigmochelys pardalis*	17.5%	15.0%	10.7%	10.9%	11.4%	11.2%	11.6%	11.9%	11.7%	11.6%	10.8%	10.8%	10.8%	10.8%	10.8%	10.8%	10.7%	10.8%	10.8%	10.8%	10.7%	10.8%	10.7%	10.6%	10.7%	10.4%	10.4%	10.1%	10.3%	10.3%	10.3%	10.0%	9.9%	10.1%	10.1%	10.0%	11.3%	11.4%	11.4%	11.4%	11.5%	10.6%	11.8%	12.1%	11.4%	14.1%	11.4%	12.3%	12.6%	12.9%	12.4%	11.8%	14.0%	13.3%	13.5%	10.6%	12.7%	12.6%	12.9%	—
61	DQ080049 *Testudo graeca* TUNISIA	17.9%	15.5%	12.9%	13.1%	13.4%	13.4%	13.4%	13.6%	13.5%	13.5%	12.7%	12.6%	12.7%	12.7%	12.7%	12.7%	12.6%	12.6%	12.6%	12.7%	12.7%	12.7%	12.6%	12.5%	12.6%	12.9%	12.9%	12.7%	12.9%	12.9%	12.9%	12.6%	12.3%	12.4%	12.7%	12.5%	12.6%	13.0%	13.0%	12.9%	13.2%	13.4%	13.9%	14.2%	13.5%	14.7%	13.8%	14.6%	10.6%	11.0%	14.4%	13.5%	12.5%	14.0%	14.1%	14.1%	15.5%	14.3%	14.5%	13.3%	—
62	DQ080050 *Testudo graeca* TURKEY	17.6%	15.4%	12.6%	12.9%	13.0%	13.2%	13.0%	13.2%	13.2%	13.2%	12.4%	12.3%	12.4%	12.4%	12.4%	12.4%	12.3%	12.3%	12.4%	12.4%	12.3%	12.3%	12.3%	12.2%	12.3%	12.4%	12.4%	12.1%	12.4%	12.4%	12.4%	12.1%	11.8%	11.8%	12.1%	12.0%	12.2%	12.4%	12.5%	12.4%	12.6%	12.8%	13.6%	13.7%	13.2%	14.4%	13.6%	14.3%	10.1%	10.6%	14.1%	13.0%	12.1%	13.7%	13.8%	13.8%	15.0%	13.9%	14.2%	12.8%	3.5%	—
63	DQ080046 *Testudo hermanni boettgeri*	18.0%	15.2%	12.2%	12.5%	12.8%	12.9%	12.8%	12.8%	12.9%	13.0%	11.8%	11.7%	11.8%	11.8%	11.8%	11.8%	11.8%	11.7%	11.8%	11.8%	11.7%	11.7%	11.7%	11.8%	11.7%	12.1%	12.1%	11.8%	12.1%	12.1%	12.1%	11.6%	11.6%	11.6%	11.7%	11.6%	12.4%	12.6%	12.6%	12.6%	12.7%	12.6%	13.6%	13.8%	13.0%	14.1%	13.2%	13.9%	9.4%	9.6%	13.8%	13.0%	11.3%	13.3%	13.3%	13.4%	14.8%	13.8%	14.2%	12.5%	10.1%	9.9%	—
64	DQ080045 *Testudo horsfieldii*	17.6%	15.5%	12.2%	12.7%	12.9%	13.2%	12.6%	12.9%	13.0%	13.0%	12.0%	11.8%	12.0%	12.0%	12.0%	12.0%	11.9%	11.8%	11.9%	11.9%	11.8%	11.8%	11.8%	11.9%	11.8%	12.3%	12.3%	12.1%	12.2%	12.2%	12.2%	11.9%	11.9%	11.8%	12.0%	11.9%	12.3%	12.5%	12.6%	12.6%	12.6%	12.9%	13.6%	13.8%	13.0%	14.1%	13.6%	14.0%	9.4%	9.5%	14.0%	13.2%	11.4%	13.4%	13.5%	13.4%	14.8%	14.0%	14.4%	12.7%	10.6%	10.2%	8.9%	—
65	DQ080048 *Testudo kleinmanni*	17.5%	15.5%	12.6%	12.9%	13.3%	13.0%	13.1%	13.5%	13.2%	13.2%	12.4%	12.4%	12.5%	12.5%	12.5%	12.5%	12.5%	12.4%	12.5%	12.5%	12.4%	12.4%	12.4%	12.3%	12.4%	12.6%	12.7%	12.4%	12.6%	12.6%	12.5%	12.3%	12.0%	12.0%	12.3%	12.3%	12.6%	12.6%	12.7%	12.6%	12.6%	13.0%	13.8%	13.8%	13.2%	14.7%	14.0%	14.4%	10.4%	10.8%	14.1%	13.3%	12.2%	13.5%	13.8%	13.7%	15.2%	14.0%	14.2%	12.9%	8.0%	7.4%	10.2%	10.3%	—
66	DQ080047 *Testudo marginata*	17.5%	15.4%	12.4%	12.8%	13.2%	13.0%	13.3%	13.5%	13.3%	13.4%	12.3%	12.3%	12.4%	12.4%	12.4%	12.4%	12.3%	12.2%	12.3%	12.3%	12.2%	12.2%	12.3%	12.2%	12.2%	12.4%	12.4%	12.1%	12.3%	12.3%	12.3%	11.9%	11.6%	11.9%	11.9%	11.9%	12.4%	12.4%	12.5%	12.3%	12.6%	12.7%	13.8%	13.8%	13.0%	14.5%	13.6%	14.3%	10.0%	10.2%	13.9%	13.2%	11.9%	13.2%	13.6%	13.6%	14.9%	13.9%	14.4%	12.9%	7.5%	7.0%	10.0%	10.0%	5.8%	—

Table S7. Genetic diversity indices and substitution rates of mitogenomes of *Chelonoidis alburyorum* and selected other testudinids based on sites without missing data/gaps.

Species	n_{seq}	n_{sites}	S	I	π	T	μ
Chelonoidis alburyorum	11	9,841	63	0	0.002	1.46 [0.91-2.21]	4.38×10^{-3}
Chelonoidis niger complex	15	13,751	304	1	0.006	2.01 [1.42-2.8]	1.10×10^{-2}
Chelonoidis denticulatus	2	13,826	33	22	0.002	0.55 [0.25-0.98]	4.34×10^{-3}
Chelonoidis carbonarius	2	13,781	240	18	0.017	3.74 [1.95-6.15]	4.66×10^{-3}
Cylindraspis spp.	5	15,203	2,022	57	0.06	26.75 [19.31-34.4]	4.97×10^{-3}
Testudo graeca	2	15,240	526	3	0.035	7.48 [4.1-11.77]	4.61×10^{-3}

n_{seq}: number of sequences; n_{sites}: total number of sites (without gaps/missing data); *S:* number of substitutions; *I:* insertions/deletions (indels); *π:* nucleotide diversity; *T*: mean and 95% HPD interval of inferred diversification time in Ma; *μ*: mean substitution rate (substitutions per site per Ma).
